# Integrating vosoritide therapy with limb surgery in paediatric patients with achondroplasia: real-life experiences

**DOI:** 10.1186/s13023-025-03853-7

**Published:** 2025-07-17

**Authors:** Anna Elsa Maria Allegri, Maria Francesca Bedeschi, Maria Beatrice Bocchi, Valentina Camurri, Michaela Veronika Gonfiantini, Chiara Leoni, Milena Mariani, Osvaldo Palmacci, Matteo Porro, Simone Riganti, Caterina Tedesco, Berardo Rinaldi, Emanuela Scarano, Concetta Schiavariello, Angelo Selicorni, Stefano Stagi, Fabio Verdoni, Giuseppe Zampino, Mohamad Maghnie, Roberta Onesimo

**Affiliations:** 1 Paediatric Endocrinology Unit, Department of Paediatrics, IRCCS Istituto Giannina Gaslini, Genoa, Italy; 2 Medical Genetic Unit, Fondazione IRCCS Ca’ Granda Ospedale Maggiore Policlinico, Milano, Italy; 3 Department of Orthopaedics and Traumatology, Fondazione Policlinico Universitario A. Gemelli IRCCS, Università Cattolica del Sacro Cuore, Roma, Italy; 4 Department of Paediatric Orthopaedics, IRCCS Giannina Gaslini, Genoa, Italy; 5 Rare Diseases and Medical Genetics Unit, Bambino Gesù Children’s Hospital, IRCCS, Rome, Italy; 6 Center for Rare Diseases, Department of Woman and Child Health and Public Health, Fondazione Policlinico Universitario A. Gemelli IRCCS, Roma, Italy; 7 Paediatric Unit ASST Lariana, Mariani foundation Center for fragile Child, Como, Italy; 8 Paediatric Physical Medicine and Rehabilitation Service, Fondazione IRCCS Ca’ Granda Ospedale Maggiore Policlinico, Milan, Italy; 9 Department of Paediatrics, IRCCS Azienda Ospedaliero-Universitaria di Bologna, Bologna, Italy; 10 Department of Health Sciences, University of Florence, Florence, Italy; 11 Meyer Children’s Hospital Scientific Institute for Research, Hospitalization and Health Care (IRCCS), Florence, Italy; 12 IRCCS Ospedale Galeazzi Sant’Ambrogio, Milan, Italy; 13 Università Cattolica del Sacro Cuore, Roma, Italy; 14 Department of Neuroscience, Rehabilitation, Ophthalmology, Genetics, Maternal and Child Health, University of Genoa, Genoa, Italy

**Keywords:** Vosoritide, Achondroplasia, Lower limb lengthening, Surgery

## Abstract

**Background:**

Achondroplasia is the most common form of disproportionate short stature and can lead to serious medical complications, including foramen magnum and spinal stenosis. Until 2021, there were no precision treatments available, and in some countries, elective surgery was considered a standard approach to increase height, improve body proportions, enhance functionality, and correct deformities in a selected group of patients. Recently, C-type natriuretic peptide (CNP) has been explored as a potential treatment, aiming to counteract the molecular activity driven by FGFR3. Although post-market and real-world data on the drug are still limited, many questions remain about the potential for combining pharmacological and surgical therapies and how this might influence patient outcomes. Concerns have also been raised regarding the potential impact of drugs on bone healing. However, anecdotal evidence from orthopaedic practice suggests that the two ossification processes do not interfere with one another. The aim of this study was to describe the first real-world case series in which vosoritide treatment was integrated with limb surgery in children and adolescents with achondroplasia.

**Results:**

Sixteen paediatric patients with molecular confirmation of achondroplasia were included in the study. All patients underwent combined vosoritide therapy and limb surgeries (13 for lower limb lengthening and 3 for varus correction through epiphysiodesis).The complementary roles of vosoritide therapy and surgery were highlighted, with treatment outcomes aligning closely with expectations.

**Conclusion:**

This report provides the first clinical description of the combination of precision therapy with limb surgery in a relatively large multicentre cohort of paediatric patients with achondroplasia. These findings support continued exploration of the integration of different therapeutic approaches.

## Introduction

Achondroplasia (ACH) (OMIM #100800) is the most prevalent type of disproportionate short stature, occurring in approximately 4.6 per 100,000 live births.

The underlying cause of ACH is a specific activating mutation of the fibroblast growth factor receptor-3 (*FGFR3*) gene at position 1138 that occurs in two variants (1138G > A and 1138G > C), resulting in the substitution of arginine with glycine at position 380 (G380R) in the transmembrane domain of the FGFR3 protein [1, 2]. 

Consequently, the activation of the downstream inhibitory signalling pathway in chondrocytes leads to impaired endochondral bone growth and significant height differences. In addition, it is associated with potentially serious medical complications, including foramen magnum and spinal stenosis, which can lead to increased morbidity and mortality [3]. In particular, faster proliferating growth plates (such as those of femurs and homers) tend to be more affected than slower ones elsewhere, leading to the characteristic rhizomelic appearance observed in individuals with achondroplasia.

The identification of the molecular mechanisms underlying ACH has generated significant interest in the pharmaceutical industry regarding this rare disease. As a result, various strategies have been developed to target the overactive FGFR3 receptor and its downstream signalling pathways, and current approaches have explored FGFR3 ligands, which block FGFR3 itself, and tyrosine kinase inhibitors that target the intracellular components of FGFR3 receptors. C-type natriuretic peptide (CNP), which acts through its receptor NPR-B, downregulates aberrant FGFR3 signalling in chondrocytes by inhibiting the MAPK-ERK signalling pathway [4]. Vosoritide, a modified form of C-type natriuretic peptide (CNP) known as the CNP-39 analogue BMN111 (BioMarin Pharmaceutical, San Rafael, Calif, USA), which has been engineered to resist degradation and increase the half-life, was first approved in August 2021 in the EU and became the first medical treatment for achondroplasia in patients aged 2 years and older. Since 2023, vosoritide has been approved for use in children aged 4 months and older until epiphyseal closure [5]. 

Several preclinical and clinical phase II and III trials have demonstrated the efficacy of vosoritide in terms of growth velocity and height Z score improvements, as well as in terms of annualized growth velocity and the upper-to-lower body segment ratio [6–8]. 

Before vosoritide approval, limb surgery was recognized as a valid treatment for increasing final height and correcting skeletal deformities (leg deformities and disproportion), significantly improving the physical condition and lower limb alignment of patients, as evidenced by an average increase of up to 30 cm in lower limb length, ultimately improving quality of life [9, 10]. However, whether and when limb lengthening surgery should be performed and which surgical methods should be adopted remain unclear [11, 12]. 

The development of medical therapy for achondroplasia has revitalized the discussion about the management of achondroplasia, both at the national and international levels.

In Italy, a group of experts discussed best practices and current unmet needs in the management of patients with achondroplasia through a two-round Delphi panel between September and November 2022, and the consensus was that a shared model for patient management should be adopted to ensure adequate continuity of care over the whole lifespan [11]. 

In the last international consensus statement on the management and lifelong care of individuals with achondroplasia, there was no mention of the use of any medical treatment, as no medical treatment had been approved at the time, so recommendations on limb surgery (correction of lower extremity malalignment and limb lengthening) vary [12]. Specific patient assessment by a multidisciplinary team should be completed before and after limb lengthening surgery is performed to consider and balance all functional, physical and psychosocial outcomes. A multidisciplinary team is necessary to achieve better outcomes and prevent complications. Procedures should be performed in well-equipped centres specializing in treatment for achondroplasia. Before performing limb lengthening surgery, whole spine and cervical and skull base magnetic resonance imaging (MRI) are mandatory to minimize the risk of spinal cord damage caused by neck extension during anaesthesia and surgery [12] and to screen for spinal stenosis [13]. 

The actual possibility of medical and surgical treatment for achondroplasia enriches treatment options for patients and even opens the possibility of a combined approach.

As there are no previous reports of the use of integrated vosoritide therapy with limb surgery (IVTLS) for the treatment of achondroplasia to date, this clinical report will fill this gap.

## Methods

The CARE (CAse REport) consensus guidelines were followed to optimize the completeness and transparency of this case series [14]. 

### Patient selection

From January 1, 2023, to February 29, 2024, a total of 16 patients diagnosed with ACH genetically confirmed at six Italian reference centres for skeletal dysplasia were retrospectively screened. The study included 16 patients who were reported to have undergone concomitant vosoritide therapy and limb surgery (limb lengthening/correction of deformities).

All patients underwent multidisciplinary evaluation in the context of multidisciplinary management of skeletal dysplasia [15, 16] and underwent orthopaedic limb surgery at three different highly specialized centres of orthopaedic surgery (Genoa, Milan, Rome). Surgery was performed according to the latest advancements in limb lengthening and deformity correction [10, 17, 18]. All types of orthopaedic surgical techniques for lower limb lengthening and correction of deformities, as well as all types of orthopaedic instruments, have been considered.

Vosoritide treatment was started according to the current European Medical Agency (EMA) and Italian Medicines Agency (AIFA) recommendations [19, 20]. 

In all centres, the same achondroplasia charts [21] were used, and standard deviation scores (SDSs) were calculated according to Merker’s reference data Table [22]. 

The main auxological and pubertal parameters, orthopaedic surgical variables, and other descriptive variables (related to the natural history and principal comorbidities or previous nonorthopaedic surgical aspects) were reported. Each patient’s previous surgical history (neurosurgery, ear, nose and throat (ENT) surgery and orthopaedic surgery) and clinical and surgical characteristics were recorded.

## Results

### Case series description

A total of 16 patients with ACH were enrolled in the study. Thirteen patients underwent limb lengthening surgery, and the remaining three patients underwent hemiepiphysiodesis surgery. Among the patients included in the case series, 15 patients started vosoritide between six months before and six months after the placement of an orthopaedic device (fixator/eight-plate/intramedullary nail). Only one patient started vosoritide after six months. Data on the orthopaedic features and adverse events during vosoritide treatment and lengthening or orthopaedic surgery are reported in Table 1.

#### Patients who underwent lower limb lengthening (*n* = 13)

##### Patient 1

A male who had undergone surgery for foramen magnum stenosis at 14 months of age and several other surgical interventions, including adenotonsillectomy and transtympanic drainage, underwent early rehabilitation and wore an orthopaedic brace for thoraco-lumbar kyphosis for three years (from 5 to 8 years old). At age 8.10 years, the patient underwent bilateral transverse lengthening of the tibiae and fibulae with a bilateral external ring fixator and bifocal corticotomy.

At age 9.2 years, the patient began therapy with vosoritide (15 µg/kg/day). At the beginning of therapy, the patient’s auxological parameters were as follows: weight 21.4 kg (< -1 SDS on the achondroplasia growth curve) and height 106.7 cm (+ 1 SDS on the achondroplasia growth curve). The upper/lower limb ratio was 1.94. BMI 19.8 kg/m2, and her head circumference was 58 cm ( < + 1 SDS on the achondroplasia growth chart), indicating Tanner stage 1. The external fixator was removed after 7 months, with a total surgical tibial lengthening of 7.7 cm.

No medical complications were observed, except for ankle stiffness due to inflammation. Therapy with vosoritide was never discontinued, and no adverse events were observed.

##### Patient 2

A female who had no previous history of neurosurgery or ENT issues during the follow-up period had a hypermobile lax interatrial septum with a small nonhemodynamically significant interatrial defect.

At age 9 years, she underwent bilateral tibial lengthening surgery, followed by bilateral femoral lengthening surgery at age 11 years. At age 13 years, she started subcutaneous therapy with vosoritide (15 µg/kg/per day); her height was 121.30 cm (+ 1 SDS on the achondroplasia growth curve), she was at Tanner stage 2, and she had not yet experienced menarche. At age 13.3 years, two weeks after the 3-month assessment, her height was 122.8 cm (+ 1 SDS on the achondroplasia growth curve), and she underwent bilateral tibia and fibula osteotomy surgery with an external fixator for bilateral tibia lengthening using the Ilizarov method. Postoperative X-rays revealed satisfactory progress in the lower limbs.

During the subsequent auxological clinical evaluation, height measurements were affected by the presence of the external fixator and the inability to load and extend the limb. X-rays performed at 60 and 180 days after surgery revealed excellent bone regeneration without any signs of pin mobilization.

Throughout the limb lengthening process, the patient continued vosoritide therapy without interruption and did not experience any significant drug-related issues, except for the expected painful symptoms associated with the surgical procedure. The external fixator was removed after 13 months, with a total surgical tibial lengthening of 6.5 cm. After 18 months of vosoritide therapy, the patient reached a height of 135.1 cm ( < + 3 SDSs on the achondroplasia growth curve). She tolerated vosoritide therapy well, and no adverse events were reported.

##### Patient 3

A male who had no previous history of neurosurgery or ENT issues during the follow-up period underwent bilateral tibia lengthening surgery and placement of an external tibia and fibula fixator at age 7 years and experienced a gain of approximately 7 cm.

At age 10.5 years, the child underwent bilateral femoral osteotomy and placement of an external fixator, which was removed after 6 months, resulting in a total height gain of 8 cm.

He started vosoritide (15 µg/kg/per day) approximately 2 months after the removal of the external fixator. The height parameters prior to therapy were within + 1/+2 SDSs on specific growth curves. During the last evaluation, after 9 months of treatment, the parameters were > + 3 SDSs above the specific growth curve. He continued vosoritide therapy and reported that he had not experienced any side effects at the last clinical evaluation; at age 11.7 years, improvements in spinal alignment and a trend towards straightening of the physiological dorsal kyphosis were observed. Furthermore, notable improvement in the limitation of left knee flexion was observed, whereas reduced muscle development was evident in the right lower limb, particularly in the calf, compared with the left side.

##### Patient 4

A female who had previously undergone surgery for foramen magnum stenosis at 2 years of age underwent surgery for bilateral tibia lengthening at age 10.5 years.

At age 12.7 years, she underwent surgery for bilateral femur lengthening (telescopic nail), and subcutaneous therapy with vosoritide (15 µg/kg/per day) was started at age 12.9 years. She had a height of 121.7 cm (+ 1.2 SDSs on the achondroplasia growth curve, -5.1 SD Z scores for the general population) and an upper/lower limb ratio of 1.5, suggesting Tanner stage 2. Therapy with vosoritide was never discontinued, and we did not observe any adverse events. At age 13.2 years, the patient underwent a new surgery in which the previous nails were replaced to allow continued lengthening. At the end, the total lengthening achieved was 10 cm (4 cm + 6 cm). Currently, considering good bone callus formation, we are planning to remove these devices in the coming months.

##### Patient 5

A male who had no previous history of neurosurgery or ENT issues during the follow-up period started subcutaneous therapy with vosoritide (15 µg/kg/per day) at age 12.7 years; the height was 117.5 cm (+ 1 SDS on the achondroplasia growth curve) and the upper/lower limb ratio was 2.1, indicating Tanner stage 2. At age 12.9 years, the patient underwent surgery for bilateral tibia lengthening and simultaneous significant genu varum correction with hexapod external fixators. The total lengthening achieved was 7 cm, with optimal angular deformity correction. Currently, patient is still being treated with external fixators while waiting for sufficient bone callus formation for their removal.

##### Patient 6

A female who had previously undergone surgery for foramen magnum stenosis at 4 years of age underwent surgery for bilateral tibia lengthening at age 7 years and bilateral femur lengthening (monoaxial external fixator) at age 12 years.

During femur lengthening, subcutaneous therapy with vosoritide (15 µg/kg/per day) was started at age 12.5 years; her height was 134.5 cm (+ 3 SDSs on the achondroplasia growth curve), her upper/lower limb ratio was 1.3, and she reached Tanner stage 2. The total femoral lengthening achieved was 8 cm. Eight months after the last surgery, considering good bone callus formation, the patient underwent external fixator removal.

Therapy with vosoritide was never discontinued, and no adverse events occurred.

##### Patient 7

A female who had previously undergone surgery for foramen magnum stenosis at age 7 months underwent adenoid surgery for obstructive sleep apnoea at age 4 years, followed by bilateral myringotomy with tube placement at age 6 years.

At age 10.6 years, she started subcutaneous therapy with vosoritide (15 µg/kg/per day); she had a height of 109.8 cm (+ 1.1 SDS on the achondroplasia growth curve, -4.7 SD Z scores of the general population), upper/lower limb ratio of 1.9, and was Tanner stage 1.

At age 10.8 years, she underwent surgery for bilateral femoral and bilateral tibia and fibula lengthening with intramedullary motorized nails, and the total lengthening achieved was 10 cm (5 cm + 5 cm). Currently, considering good bone callus formation, we are planning to remove these devices in the coming months.

No adverse events were reported. Therapy with vosoritide was never discontinued, with no adverse events.

##### Patient 8

A male who had no previous history of neurosurgery or ENT issues during the follow-up period developed obesity and exhibited poor glucose tolerance at age 12 years and started treatment with metformin (500 mg, twice a day).

At age 13 years, his height was 118.8 cm (-5.29 SD), weight was 31.6 kg, and BMI was 22.39 (0.61 SD) according to Italian general population growth charts. At age 13.4 years, he underwent femur lengthening surgery and started treatment with vosoritide (15 µg/kg/day) one month later, with no adverse events. At the time of orthopaedic surgery, his height was 120.7 cm (-5.13 SD), and his BMI was 22.24 (0.48 SD); furthermore, at the time of the start of vosoritide treatment, his height was 121.9 (-4.94 SD), his BMI was 20.71 (0.02 SD), his upper/lower limb ratio was 1.7, and his bone age was 14.7 years. He was in pubertal stage 3 according to the Tanner scale.

At age 13.9 years, his height was 129.4 cm (-4.15 SD), and his BMI was 22.34 (0.44 SD). The height gain between surgery and the end of orthopaedic treatment was 8.7 cm (0.98 SD), whereas the gain between the start of vosoritide therapy and the end of orthopaedic treatment was 7.5 cm (0.79 SD).

The vosoritide treatment ended when the near adult height was reached at 14.8 years: the height was 130.5 cm (-4.49 SD), the BMI was 41.4 kg, the BMI was 24.31 (0.88 SD), the upper/lower limb ratio was 1.4, and the height velocity was 1.1 cm/year. He was in pubertal stage 4 according to the Tanner scale. The patient’s bone age was 16.3 years.

##### Patient 9

A female who had no previous history of neurosurgery or ENT issues during the follow-up period underwent bilateral tibial surgery and varus correction with external fixators at age 10.7 years (height 110.5 cm, + 0.19 SDS). The lengthening procedure was performed 1 week after surgery (fixator position), and the fixators remained in place for a total duration of 2.8 months. The fixators were removed 9 months after their positioning.

On the 35th day after external fixator removal, when the patient was 11.5 years, subcutaneous therapy with vosoritide (12.5 mcg/kg/per day) was started; the height was 119.5 cm (+ 1.17 SDS).

The height gain was 9 cm, which was related to lengthening alone before starting vosoritide. She was in pubertal stage 2 according to the Tanner scale, and no adverse events were reported.

##### Patient 10

A male who had no previous history of neurosurgery or ENT issues during the follow-up period underwent bilateral femoral surgery and varus correction with external fixators at age 13.4 years (height 110.5 cm, -1.94 SDS). He started lengthening on postoperative day 7 and continued lengthening for 100 days, achieving a height of 117.2 cm (-0.6 SDS) according to achondroplasia growth charts.

Forty-two days after the completion of lengthening, subcutaneous therapy with vosoritide was started at a dose of 13.55 mcg/kg/day and not discontinued, and no adverse events were observed; he was in pubertal stage 1 according to the Tanner scale.

##### Patient 11

A male who had previously undergone surgery for foramen magnum stenosis at 14 months of age presented with obstructive sleep apnoea, recurrent otitis, and conductive hearing loss, which resolved with adenotonsillectomy and transtympanic drainage between 3 and 5 years of age.

A ventricular septal defect was found incidentally at birth and surgically corrected at age 40 days, without cardiological sequelae, and a significant varus deformity of the right tibia was observed early in life.

At age 11 years, he underwent bilateral tibial surgery and varus correction with external fixators; his height was 105.2 cm (-1 SDS).

At age 11.1 years (height 105.2 cm, -1 SDS), lengthening started 7 days after surgery, lasting for 98 days, and led to a height of 116.2 cm (-1 SDS) at 11.1 years of age.

At age 11.5 years, vosoritide therapy was started at a dosage of 15.87 µg/kg/day, 56 days after the end of lengthening, and not discontinued, and no adverse events occurred. At the time of the clinical evaluation, the patient had a height of 114.2 cm, a ± 0.12 SDS and in pubertal stage 2 according to the Tanner scale.

##### Patient 12

A male who had no previous history of neurosurgical problems developed obstructive sleep apnoea, recurrent otitis and conductive hearing loss, which resolved with adenotonsillectomy and transthympanic drainage between 8 and 9 years of age, with persistent mild hearing impairment.

Orthopaedic surgery with femoral lengthening was performed at 11.8 years of age, and fixators were removed after 8.4 months. Subcutaneous therapy with vosoritide started at age 13.6 years (12.8 µg/kg/per day); the patient had a height of 128.4 cm (+ 1.64 SDS) and was in pubertal stage 3 according to the Tanner scale. After 88 days of therapy, a second tibial and peroneal lengthening surgery with an external fixator was performed. Vosoritide therapy was discontinued at the start of surgery and during the initial lengthening phase to avoid therapy overcharge during the period in which the patient was taking pain relievers (AINEs, acetaminophen and opioids) and benzodiazepines.

The second lengthening surgery was needed because of surgical bone callus rupture due to early bone consolidation. Vosoritide therapy was discontinued because of the need for additional analgesic therapy. Vosoritide was restarted thereafter at a dose of 12.7 µg/kg/day, with no adverse events.

##### Patient 13

A female with a medical history of recurrent otitis and obstructive sleep apnoea was treated with continuous positive airway pressure (CPAP) and foramen magnum decompression at age 8 years.

At age 9 years, she underwent surgery for bilateral tibia lengthening with a circular external fixator, resulting in a height gain of 7 cm.

Vosoritide therapy was started at age 12.4 years (15 µg/kg/per day), at Tanner stage 4, with a height of 111.5 cm (M to -1 SDS of achondroplasia growth curves). Therapy was not discontinued, and no adverse events occurred. At age 12.7 years, the patient underwent surgery for bilateral femur lengthening with monoaxial external fixators. The total femoral lengthening achieved was 8 cm. Currently, patient is still being treated with external fixators while waiting for sufficient bone callus formation for removal. Therapy with vosoritide was not discontinued during surgical treatment, and no vosoritide-related adverse events were reported during the combined treatment. At age 13.1 years, the patient reached a height of 122 cm.

#### Patients who underwent varus correction via epiphysiodesis (*n* = 3)

##### Patient 14

A male who had previously undergone adenoid surgery for obstructive sleep apnoea at 3 years of age underwent foramen magnum stenosis decompression two times, at the ages of 7 years and 9 years.

At age 11.7 years, he underwent surgery for correction of genu varum (eight plates), and subcutaneous therapy with vosoritide (15 µg/kg/per day) was started at age 11.7 years, two days before orthopaedic correction, and not discontinued; the limb lengthening procedure was refused by the family for surgical treatment.

At the beginning of therapy, the patient had a height of 111.9 cm (+ 2 SDSs on the achondroplasia growth curve), an upper/lower limb ratio of 1.7, and was Tanner stage 1, no adverse events were reported.

##### Patient 15

A female who had previously undergone surgery for foramen magnum stenosis at 8 months of age underwent right tibia lateral hemiepiphysiodesis surgery for varus correction with eight plates at age 10 years.

Vosoritide therapy was started at age 10.2 years, 60 days after orthopaedic surgery, at a dosage of 15.3 µg/kg/per day. At the time of the clinical evaluation, the patient had a height of 105.5 cm (-0.04 SDS) and was between pubertal stages 1 and 2 according to the Tanner scale. Therapy with vosoritide was never discontinued. To date, we have observed no adverse events.

##### Patient 16

A male who had previously undergone surgery for foramen magnum stenosis at 6 months of age underwent adenotonsillectomy for ENT problems at 3.5 years of age. At 1.5 years of age, the patient was diagnosed with severe obstructive sleep apnoea syndrome and recommended for nighttime support with CPAP.

In addition, between 5 and 6 years of age, acetazolamide therapy was started because of intrabulbar bulging observed on magnetic resonance images and was discontinued after the exclusion of endocranial hypertension. At age 6 years, he was diagnosed with hypertension, which requires treatment with amlodipine.

At 8.5 years of age, he underwent correction of a lower extremity varus deformity with lateral hemiepiphysiodesis with plaque at the level of the knees bilaterally (height 106.5 cm, + 0.79 SDS). Vosoritide therapy was started 92 days after orthopaedic surgery at a dose of 12.8 µg/kg/per and was not discontinued, no adverse events occurred; at the time of the clinical evaluation, the patient had a height of 108.7 cm (+ 1.3 SDS) and was in pubertal stage 1 according to the Tanner scale.

### b - Cohort summary and treatment overview

Sixteen paediatric patients with molecular confirmation of achondroplasia was treated with IVTLS (13 for lower limb lengthening and 3 for varus correction through epiphysiodesis).

The median age of this cohort was 12.6 ± 1.0 years (females = N°7; males = N°9). Six out of the 13 patients underwent IVTLS at the tibial level exclusively, and 6 out of the 13 patients underwent IVTLS at the femoral level exclusively, whereas the last patient underwent IVTLS at both the femoral and tibial levels with intramedullary nails.

Eight out of the 13 patients previously underwent limb surgery before vosoritide was approved for use.

One of the 16 patients discontinued vosoritide therapy at the start of surgery and during the initial lengthening phase to avoid analgesic overuse.

In our cohort, a total of 9 patients started treatment with vosoritide within six months before orthopaedic surgical treatment, six underwent surgical procedures while receiving vosoritide treatment, and only one underwent orthopaedic limb surgery more than six months before starting treatment with vosoritide.

The combination of precision therapy and surgery did not cause any deviations from the expected treatment outcome.

## Discussion

This case series study, which was conducted by an Italian multidisciplinary, multicentre team, examines information collected from a group of ACH patients treated with IVTLS to provide real-life experiences in treating such patients.

Information from 51 paediatric patients were prospectively included in the ACH registry over a 6-year period. Only one patient underwent orthopaedic limb lengthening during treatment [23], whereas patients who underwent bone surgery were excluded from the phase III trial [7]. 

This study has made it possible to lay some foundations that will hopefully be useful in the future to fill the gap in real-world data or clinical studies addressing the use of vosoritide and its potential integration with limb surgery.

To date, most considerations are based on experience and beliefs, and there are no data to support or discredit the combined approach [24]. However, the expert panel expressed strong opinions, all of which were in line with recent guidance [12, 25], that both treatments should be offered in specialist centres with multidisciplinary experience in treating achondroplasia and that having both treatments available in one place is essential to enable effective analysis of a combined approach.

Studies in mice have demonstrated that the activation of guanylyl cyclase (GC)-B stimulates chondrocyte proliferation and hypertrophy in the growth plate of long bones, with increased trabecular bone volume fraction (BV/TV), trabecular bone mineral density (BMD), and cortical cross-sectional area and thickness, which results in stiffer and stronger bones.

These results provide the foundation for future studies examining how GC-B increases osteoblast numbers and/or activity as well as whether CNP analogues such as BMN-111, which play a role in GC-B activation, can effectively treat osteoporosis with a possible role in the quality of bone.

These aspects concerning bone health are extremely important points to be evaluated in the future, especially in cases of IVTLS, in which both endochondral and membranous ossification are involved [26, 27]. 

The data that emerged from this description of cases allowed us to conclude that, despite the short observation period, there were no significant changes during lengthening and medical therapy and that the starting data should be standardized as much as possible at the beginning of the combined therapy to be able to optimize the future reading of the long-term outcomes of the combined therapy itself, considering the possible emerging role of basic research studies of medical therapy on bone quality.

The median age of our cohort was 12.6 ± 1.0 years, indicating that the patients in our cohort were older than those in previously published studies [6, 8] and registries [23, 28]. 

We observed differences in surgical treatment modality, supporting data reported in international and European consensus statements.

Vosoritide has demonstrated improved efficacy in patients who adhere to a schedule of daily subcutaneous injections, minimizing breaks or discontinuations [7, 29, 30]. 

To achieve maximum therapeutic continuity despite surgical lengthening, in most of the presented.

cases except one, vosoritide was not discontinued during the perioperative period.

Additionally, excessive use of drugs related to surgery, such as analgesics, anti-inflammatories, and anaesthetics, especially during daily subcutaneous injection therapy, has gained increasing attention from multidisciplinary teams; however, to date, there are no data on the effects of pausing treatment while patients are undergoing surgery.

However, there is potential for antibody production if vosoritide is administered in a stop-start manner, but there are no data to support or contradict this hypothesis.

A reduced impact on the final height may also be observed, and pausing vosoritide may create psychosocial stress for young individuals.

In this cohort, the possible side effects known during medical therapy alone and during orthopaedic surgery alone were considered separately without observing changes with respect to the usual medical and surgical complications observed.

No serious systemic adverse effects were detected during treatment with vosoritide (vomiting, headache, and a decrease in blood pressure); 7 patients (43%) reported at least one local adverse event, such as transient reactions at the injection site and erythema, confirming that the EMA reported that the most common adverse reactions to vosoritide were injection site reactions (85%), vomiting (27%), and a reduction in blood pressure (13%) [31]. 

In practice, the use of limb lengthening varies by geographic area and may be associated with a high treatment burden and serious complications. In one of the largest case series published on limb lengthening with the Ilizarov technique, approximately 45% of patients experienced perioperative complications [9]. 

Our data revealed that neither intraoperative nor postoperative complications occurred in surgical patients.

We have no data concerning bone quality, but this reassuring observation could be related to the fact that these two therapeutic options target different regions of long bones, as vosorotide acts on proliferating chondrocytes at the epiphyseal growth plate, whereas surgery targets the metaphysis. Moreover, whenever the fracture edges are stable, ossification occurs mostly by intramembranous bone formation, a distinct mechanism compared with endochondral ossification typical of growth plates [32]. 

This last point needs careful longitudinal observation of bone quality outcomes after IVTLS to assess data regarding the pathophysiology and natural history of long bone pathology [33] in achondroplasia patients and new emerging data after the introduction of medical therapy not only in humans but also in mouse models [26, 27]. 

For example, it should be emphasized that with the introduction of medical treatment (vosoritide, infigratinib), the prevalence of enchondromas in children with achondroplasia seems to be higher than the approximately 5% previously suggested [34, 35]. 

The aetiology of enchondromas in achondroplasia patients is thought to be due to inhibition of chondrocyte maturation, and it is uncertain how (or even if) these new drugs increase the incidence of enchondromas or if there is an increase in the detection rate, as radiographs were previously not routinely performed in achondroplasia patients.

Additionally, this clinical observation study highlights the differences in clinical and surgical practices, with the goal of standardizing them for the most accurate possible reading of long-term outcomes.

Furthermore, since previous studies have revealed that achondroplasia is associated with multisystem complications, reduced quality of life and functionality, and increased pain [32, 36], it will be interesting to understand what type of impact the combined therapy of vosoritide and limb lengthening will have on these features.

Although this remains a preliminary and limited experience, vosoritide and lengthening surgery could represent a future strategy in the treatment of achondroplasia in most cases.

Whether this combination represents an advantage in terms of potential improvement in the quality of life of patients, further improvement in postural and neuromotor development or callus formation/bone quality is needed.

This case series has several limitations.

First, the data were reported in an ad hoc format shared between participating centres, which could introduce the risk of structural and reporting biases among participants. We emphasize that, in each Expert Centre, accurate height measurement was difficult due to the presence of fixators or due to forced postures in the functional recovery phase, which prevented the correct positioning of the patient during the measurement processes. Furthermore, since many patients were recently enrolled, medium- and long-term data are not available.

According to the published data, no deviations from the usual course were observed in either treatment. The surgical outcome remains unchanged, and the therapeutic outcome is also unaffected. Nonetheless, the authors strongly recommend prospective data collection to fill these gaps, ensuring dataset uniformity, and offering valuable insights into the long-term efficacy of combination therapy. Future prospective studies with standardized protocols are essential to provide comprehensive information on the long-term safety and effectiveness of combination therapy.

Considering improved access to new therapies, both limb lengthening and corrective procedures, as well as pharmacological options, these therapeutic alternatives will become increasingly integrated over time.

In this series, we explored lower limb lengthening. Humeral lengthening, which significantly affects autonomy, is less often discussed and lacks data. The use of noninvasive lengthening methods and early vosoritide treatment may reduce the need for surgery. This paper is essential for guiding management and future data collection to gather more evidence.

This was the third workshop in this BOND-ERN ISCB series, and 138 delegates and 12 faculty members (total of 150) attended the workshop from 31 countries and 71 different cities.

The aim of this workshop was to present medical and orthopaedic teams specializing in the pathophysiology of long bone pathology to raise awareness of the orthopaedic options available to patients and explore the impacts of some of these procedures on the functional status of these patients, as well as their personal experiences.

## Conclusion

This study evaluates the efficacy of vosoritide treatment combined with limb surgery in a large cohort of achondroplasia paediatric patients. Additional large, prospective studies with accurate, long-term data are needed before definitive conclusions can be drawn.

**Table 1:** Orthopedic features and adverse events during Vosoritide treatment and lenghtening or orthopedic surgery

	Case 1	Case 2	Case 3	Case 4	Case 5	Case 6	Case 7	Case 8	Case 9	Case 10	Case 11	Case 12	Case 13	Case 14	Case 15	Case 16
**Sex**	M	F	M	F	M	F	F	M	F	M	M	M	F	M	F	M
**Age (yrs) start vosoritide**	9.2	13.	11.2	12.9	12.7	12.5	10.6	13.5	11.5	13.5	11.5	13.6	12.4	11.7	10.2	8.8
**Age (yrs) start limb lengthening/orthopedic surgery**	8.10	13.3	10.5	12.7	12.9	12	10.8	13.4	10.7	13.4	11	13.9	12.7	11.7	10	8.5
**Time of treatment Vosoritide (months)**	10	12	13	11	9	9	12	12	6	8	6	9	8.5	9	14	7
**Orthopedic surgery performed**	Limb Lenghtening	Limb Lenghtening	Limb Lenghtening	Limb Lenghtening	Limb Lenghtening and varus correction	Limb Lenghtening	Limb Lenghtening	Limb Lenghtening	-	Limb Lengthening	-	Limb Lengthening	Limb lenghtening	Hemiepiphysiodesis in genu varum	hemiepiphysiodesis surgery	lateral hemiepiphysiodesis with plaque
**Number of limb lengthening**	1	2	2	2	1	2	2	2	1	1	1	two osteotomies	2	0	0	0
**Bone Segment lengthened**	Bilateral Tibias	Bilateral Femurs	Bilateral Femurs	Bilateral Femurs	Bilateral Tibias	Bilateral Femurs	Bilateral Femurs and Tibias	Bilateral Femurs	Bilateral Tibias	Bilateral Femur	Bilateral Tibias	Bilateral Tibias	Bilateral Femurs	none	none	none
**Orthopedic device**	Circular external fixator	Axial external fixator	Axial external fixator	Intramedullary lengthening system	Hexapod External Fixator	Axial external fixator	Intramedullary lengthening system	Axial external fixator	Circular external fixator	Axial external fixator	Circular external fixator	Circular external fixator	Axial external fixator	Eight Plate	right lateral proximal tibia eight plate	-
**Discontinuation of vosoritide **	no	no	no	no	no	no	no	no	no	no	no	yes	no	no	no	no
**Orthopedic Device Time(months)**	7	13	9	12	6	8	10	8	12	11	10	11	5	-	-	-
**Adjuvanted therapies for pain treatment**	Ibuprofen	Ibuprofen	Ibuprofen	Paracetamol Ibuprofen	Paracetamol Ibuprofen	Paracetamol Ibuprofen	Ibuprofen	Paracetamol Ibuprofen Opioids	NSAIDs Opioids acetaminophen benzodiazepines	NSAIDs Opioids acetaminophen benzodiazepines	NSAIDs Opioids acetaminophen benzodiazepines	NSAIDs Opioids acetaminophen benzodiazepines	Paracetamol Ibuprofen	Paracetamol Ibuprofen	NSAIDs acetaminophen	NSAIDs acetaminophen
**Complication related surgery (yes/no)**	yes	no	no	no	no	no	no	no	Mild supeficial skin infections	Superficial skin infections	-	Surgery was performed twice due to premature anda delayed bone consolidation	no	no	-	-
**Superficial infections**	no	no	no	no	no	no	no		no	yes	no	no	no	no	not applicable	not applicable
**Deep Infections**	no	no	no	no	no	no	no	no	no	no	no	no	no	no	not applicable	not applicable
Delayed Union*	yes	no	no	no	no	no	no	no	no	no	no	no	no	no	not applicable	not applicable
**Fractures**	no	no	no	no	no	no	no	no	no	no	no	no	no	no	not applicable	not applicable
**Other**	no	no	no	no	no	no	no	no	no				no	no	not applicable	not applicable
**Adverse events of Vosorotide treatment**	no	no	no	no	no	no	no	yes	yes	yes	yes	yes	no	no	yes	yes
**Injection site reaction**	no	no	no	no	no	no	no	yes	yes	yes	yes	yes	no	no	yes	yes
**Vomiting**	no	no	no	no	no	no	no	no	no	no	no	no	no	no	no	no
**Headache**	no	no	no	no	no	no	no	no	no	no	no	no	no	no	no	no
**Blood pressure decreased**	no	no	no	no	no	no	no	no	no	no	no	no	no	no	no	no

**Legend NSAIDs** Non-Steroidal Antiinfiammatory Drugs

## Data Availability

All data supporting the findings of this study are available within the paper. Declaration from https://www.springernature.com/gp/authors/research-data-policy/data-availability-statements.
